# PROTOCOL: Organised crime groups: A systematic review of individual‐level risk factors related to recruitment

**DOI:** 10.1002/cl2.1022

**Published:** 2019-07-23

**Authors:** Francesco Calderoni, Elisa Superchi, Tommaso Comunale, Gian Maria Campedelli, Martina Marchesi, Niccolò Frualdo

**Affiliations:** ^1^ Transcrime, Università Cattolica del Sacro Cuore Milan Italy; ^2^ Dipartimento di Sociologia e Ricerca Sociale Università degli Studi Milano Milan Italy

## 1 BACKGROUND

### 1.1 The issue: organised crime

Organised crime (OC) has a detrimental impact on many countries all over the world. Globalisation has facilitated the flow of people, goods, and capital, and criminal organisations have proven to be equally mobile (Adamoli, Di Nicola, Savona, & Zoffi, 1998; Morselli, Turcotte, & Tenti, 2011; Passas, 1999; Varese, 2011). Research on OC originated in the United States during the 20th century (Woodiwiss, 2003). American scholars mainly focused on the Italian–American mafias (Abadinsky, 1981; Albini, 1971; Block & Scarpitti, 1985; Cressey, 1969) and drug trafficking organisations (DTOs). In Europe, studies focused on the Italian mafias (Gambetta, 1993; Paoli, 2003), but also on organised crime groups (OCGs) from other ethnic backgrounds and countries (Fijnaut & Paoli, 2004b; Varese, 2005). In Asia, scholars particularly examined the Chinese Triads and the Japanese Yakuza (Chu, 2000; Hill, 2003; Kaplan & Dubro, 2003). More recently, researchers analysed OCGs in Latin America, with a particular attention to the development of DTOs (Bagley & Rosen, [Link]; Bagley, 2004; Beittel, Chambers & Hale, 2012; Bunker, 2015; de la Miyar, 2016; Vásquez, 2015). Overall, studies on OC encompass a variety of countries and criminal organisations, making this field of study particularly complex due to the different socioeconomic and cultural conditions.

The differences in the study of OC have inevitably influenced the challenge of defining and conceptualising OC, which has long been debated in academia and beyond (Calderoni, 2012; Finckenauer, 2005; Hagan, 1983, 2006; Symeonidou‐Kastanidou, 2007; Von Lampe, 2008, 2015). The term “organised crime” first emerged in the late 19th century in the United States, but its meaning varied over the past century (Fijnaut & Paoli, 2004a; Kenney & Finckenauer, 1995). OC was first associated with activities protected by public officials (e.g., prostitution and racketeering), and subsequently also with fraud and extortion (Woodiwiss, 2003). In the 1950s, the concept evolved towards the “alien conspiracy” approach, due to the influence of the media and US institutions such as the Kefauver Committee. The alien conspiracy approach contended that OC was predominantly composed of foreign, especially Italian immigrants, criminals organised in formally hierarchical groups and dominating profitable illegal markets such as gambling, prostitution and narcotics (Cressey, 1969). By the 1960s, several scholars rejected this approach, suggesting that OC mostly revolves on social connections, patron–client relationships and the social organisation of the underworld (Albini, 1971; Blok, 1974; Hess, 1973/1973; Ianni & Reuss‐Ianni, 1972; Smith, 1975). In the 1970s, the paradigm of the “illegal enterprise” replaced the alien conspiracy, shifting the focus on the role of criminal organisations in supplying illegal products and services (Arlacchi, 1983; Block, 1980/1983; Reuter, 1983; Smith, 1975). A particular theoretical interpretation contended that OC specialises in the supply of illegal protection (Gambetta, 1993; Varese, 2005, 2010). The economic perspective became equally predominant in Europe, which had largely remained out of the debate until the mid‐1970s (Fijnaut & Paoli, 2004a). Ever since, the OC label has become increasingly popular all over the world, and authors have proposed a variety of definitions (Von Lampe, 2016).

Notwithstanding several shifts in the conceptualisation of OC, the theoretical debate has so far failed to achieve an agreement on its definition. Several studies reviewed existing definitions to identify common dimensions (Finckenauer, 2005; Hagan, 1983; Hagan, 2006; Maltz, 1976; Van Duyne, 2004; Von Lampe, Van Dijck, Hornsby, Markina, & Verpoest, 2006). These efforts yielded several conclusions. First, the problematic element in the concept of OC is the term “organised” and its operationalisation. Consequently, most interpretations attempted to distinguish OC from “crimes that are organised”, that is, complex criminal activities requiring important levels of coordination among the participants but lacking the additional features of OC (Finckenauer, 2005). Second, it is important to distinguish between the characteristics of the group and those of the crimes and activities it perpetrates. When considering the groups, OC should be conceptualised as an ordinal rather than a binary category, with groups exhibiting several elements continuum rather than a threshold (Hagan, 1983, 2006, p. 200). Third, notwithstanding the heterogeneity in the literature, most contributions identify a core set of dimensions of OC and namely: (a) Its nonideological nature, that is, OCGs do not have political or religious motivations; (b) OC is profit oriented, aiming to achieve illegal profits; (c) continuity, that is, OC aims at the repeated commission of an indeterminate number of crimes; (d) OC uses threat and violence to perpetrate crimes; (e) OC has an internal organisation, not necessarily a formal hierarchy, such as a division of tasks; and (f) OC is embedded in the surrounding social environment and actively interacts with it, for example, by corrupting public officials, providing extra‐legal protection, controlling legal activities, influencing politics. While the attempts to define OC share important similarities, some scholars have contended that the very concept of OC is problematic and the result of a social construct rather than a useful tool for empirical analysis (Van Duyne, 1995; Von Lampe et al., 2006). Notwithstanding these criticisms, OC has remained a popular concept both in the scholarly literature and in the general public discussion.

This systematic review relies on the definition provided by Article 2 of the United Nations Convention against Transnational Organised Crime (United Nations, 2000):

*“Organized criminal group” shall mean a structured group of three or more persons, existing for a period of time and acting in concert with the aim of committing one or more serious crimes or offences established in accordance with this Convention, in order to obtain, directly or indirectly, a financial or other material benefit.*

The UN Convention definition is the result of international efforts in stepping up the fight against criminal organisations in the 1990s. Although it has been criticised for being excessively vague (Calderoni, 2012; McClean, 2007; Paoli, 2014), the UN definition suits the purposes of this systematic review by providing a broad, inclusive, operationalisation of OC. This allows for more flexibility when searching for potentially relevant studies, encompassing a variety of OCGs as the mafias, drug trafficking groups, and some criminal gangs.

### 1.2 Recruitment into OC

This systematic review aims at summarising and consolidating the knowledge on the factors associated with recruitment into OC. Entering into an OCG is a significant step in the life of an individual, constituting a negative turning point in life and determining an increase in the risk of offending, harm and incarceration (Laub & Sampson, 1993; Melde & Esbensen, 2011). Furthermore, individuals involved in OCGs are responsible for serious crimes with wide‐ranging societal implications, including loss of lives, economic impact and politics (Lavezzi, 2008; Pinotti, 2015). For the purpose of this review, recruitment refers to the different processes leading individuals to the stable involvement into OCGs. This interpretation comprises individuals deliberately choosing to participate in criminal organisations, but also subjects socialised into criminal groups through family, friendship, and community relations. It also includes, but it is not limited to, the processes of formal or ritual affiliation exhibited by some OCG (which would unnecessarily restrict the scope of the review were they adopted as operational definition). Conversely, this definition excludes individuals occasionally cooperating or co‐offending with members of OCGs, as they lack stability over time.

### 1.3 The risk factors for recruitment into OC

Criminological studies have long focused on differences in offending patterns between individuals rather than on risk factors or changes in offending patterns within individuals over time (Farrington, 2003). Nonetheless, scholars have recently turned to a risk‐factor approach to identify the factors that lead individuals to join delinquent groups and OCGs within the society they belong to. This process has been mainly driven by the expansion of developmental and life‐course criminology during the 1990s (Farrington, 2003, p. 222; Kleemans & De Poot, 2008).^1^ Several researchers have addressed changes in offending patterns within individuals engaged in OC (Kleemans & De Poot, 2008; Morselli & Tremblay, 2004; Morselli, 2003; Van Koppen, de Poot, & Blokland, 2010; Van Koppen, Poot, de Kleemans, & Nieuwbeerta, 2010), while others have taken a closer look at risk factors for joining OCGs (Kleemans & De Poot, 2008; Kleemans & Van de Bunt, 1999; Kleemans & Van Koppen, 2014; Klein & Maxson, 2006; Lyman & Potter, 2006). In this regard, some scholars have focused on the importance that social relations may play (Cornish & Clarke, 2002; Kleemans & De Poot, 2008; Kleemans & Van Koppen, 2014), while others have drawn more attention on economic disadvantages (Carvalho & Soares, 2016; Lavezzi, 2008, 2014).

### 1.4 How the risk factors may impact the recruitment into OCGs

Organised criminals do not operate in a vacuum, but they are embedded in social environments. Social factors may play a major role in OC, more than in other forms of crime. This would depend on the specific aspects distinguishing OCGs from lesser organised forms of crime: (a) Their transnational nature, (b) the importance of social relations, and (c) the need for several co‐offenders and specific expertise for the complexity of the activities conducted (Cornish & Clarke, 2002; Kleemans & De Poot, 2008). All in all, social ties with co‐offenders and with the legal world may constitute a crucial aspect for the success of OC‐related activities. Moreover, some psychological disorders, for example, substance abuse disorders, low self‐control, and/or history of past disorders and negative development, may also serve as an explanation for involvement into OC. Possible facilitators in the emergence of OCGs may also be inequality, impairments to the rule of law, and the presence of illegal and informal markets (Bandiera, 2003).

### 1.5 Why it is important to do this review

A better understanding of the factors associated with recruitment into OCGs is needed to improve and consolidate the knowledge of OC, and to design empirically based prevention strategies. For this purpose, this systematic review aims at summarising the existing empirical evidence about the relative strength of the risk factors related to recruitment into OCGs. The theoretical debate on the definition of OC has often neglected empirical research. To the best of our knowledge, there are no systematic reviews on OC, except for meta‐theoretical classifications and content analysis of definitions (Hagan, 2006; Von Lampe et al., 2006). While only partially overlapping with OC literature, gang research has produced a few systematic reviews. Previous systematic reviews have focused on youth gang membership and interventions (Hodgkinson et al., 2009; Klein & Maxson, 2006; Raby & Jones, 2016). The Campbell Collaboration has published three systematic reviews on the involvement of young people in gangs (Fisher, Montgomery, & Gardner, 2008a; 2008b; Higginson et al., 2015), and more recently one on predictors of youth gang membership in low‐ and middle‐income countries (Higginson et al., 2018). Furthermore, a parallel review on the factors leading to radicalisation and recruitment into terrorism has been registered with the Campbell Collaboration (Litmanovitz, Weisburd, Hasisi, & Wolfowicz, 2017). While these reviews show the growing interest for the risk factors leading to involvement into criminal groups, they did not consider the factors relating to recruitment in other types of groups, namely OCGs.

Several scholars addressed the importance of the social environment for the individual involvement in OC (Kleemans & De Poot, 2008; Kleemans & Van de Bunt, 1999; Kleemans & Van Koppen, 2014; Morselli, 2009; Van Koppen, de Poot, et al., 2010). Notwithstanding the growing interest in the social embeddedness of organised criminals, knowledge about the processes that lead individuals to join OCGs is widely dispersed. This systematic review therefore aims at providing a comprehensive overview of the current knowledge on the risk factors for recruitment into OCGs.

A systematic and scientific approach on empirically based findings will provide a better understanding of OC. This review aims to inform not only the academic literature on the factors associated with recruitment into OCGs, but also to be helpful for the formulation of effective evidence‐based intervention and prevention policies. By identifying the most important factors of pathways to OC membership, this review seeks to provide policy makers with detailed information on how to design potential intervention strategies. The importance of proper prevention policies against OC links to the fact that arrests only cause temporary drawbacks to the functioning of OCGs. In fact, their resilience to law enforcement interventions is one of the most distinct features of OCGs. This is due to OCGs ability to rapidly reorganise and to easily recruit new members. From an opportunity reduction perspective, intervention within the recruitment process could be an effective complementary strategy for combating OC. In this regard, the results of this systematic review may be used to inform about the most common risk factors for recruitment into OCGs, and hence to develop intervention strategies mitigating these factors. Finally, the findings may provide policy makers with more comparative insights about the dynamics of recruitment into various OCGs. Shedding light on similarities in pathways into OC may help to formulate effective criminal justice policies applicable in various countries.

## 2 OBJECTIVES

This systematic review has two main objectives:

- Objective 1: Summarise the empirical evidence on the risk factors associated with the recruitment into OCG.
- Objective 2: Assess the relative strength of the risk factors across different types of factors, types of OCGs, and countries.

## 3 METHODOLOGY

### 3.1 Criteria for including and excluding studies

#### 3.1.1 Study design

This systematic review aims at identifying and evaluating existing knowledge on the risk factors leading to recruitment to OCGs. Because recruitment into OC cannot be the object of experimental interventions, experimental and quasi‐experimental studies are not relevant to the aim of this systematic review. This review will examine empirical evidence resulting only from studies using an observational research design.

To be included, studies must report on recruitment into OCGs as one of the main objectives of the analysis, and provide details on the sampling strategy, data collection and the type of analysis conducted, that is, the relation between a risk factor and recruitment into OCG. This review will exclude literature reviews, theoretical and conceptual contributions and editorial pieces. Based on the recommendations of the anonymous reviewers and of our understanding of the field, this systematic review will retrieve and screen both quantitative and qualitative studies. Quantitative studies will undergo the selection process described in the Statistical Procedures subsection. Qualitative studies will be systematically retrieved, screened for inclusion and coded. In accordance with current Campbell Collaboration policy on systematic reviews, they will be used to inform and contextualise the evidence and findings of the quantitative studies.

For quantitative synthesis, we will rely on studies with variability in recruitment into OC, measuring and comparing at least two groups (e.g., OC prisoners and non‐OC prisoners). The review will include studies based on longitudinal and cross‐sectional designs. To be included in a meta‐analysis, each study must report at least an effect size, or allow calculation of an effect size based on the information provided.

We will not exclude studies based on their geographical scope or year of publication. In addition, we will not exclude studies based on their quality. We will evaluate the risk of bias resulting from study quality using a risk‐of‐bias tool adapted from Higginson et al. (2018) recent Campbell systematic review and PROBAST tool for prediction studies (see below, Quality assessment subsection).

#### 3.1.2 Types of OCGs

As discussed in Section 1, the definition of OC has generated a long‐lasting debate in the literature. To favour inclusion of the largest number of possible studies, this systematic review will rely on the definition provided by Article 2 of the United Nations Convention against Transnational Organized Crime (United Nations, 2000):

*“Organized criminal” group shall mean a structured group of three or more persons, existing for a period of time and acting in concert with the aim of committing one or more serious crimes or offences established in accordance with this Convention, in order to obtain, directly or indirectly, a financial or other material benefit.*

This definition includes a variety of OCGs, ranging from traditional mafias to DTOs and adult gangs. Given the important share of adult offenders in OC and the relevance of the ties to the legitimate world, the systematic review will exclude youth (street) gangs, prison gangs and terrorist groups. The literature generally considers youth street gangs as different from OCGs (Decker & Pyrooz, 2014). Furthermore, recent systematic reviews have already assessed the factors leading to youth gang membership (Higginson et al., 2018; Klein & Maxson, 2006). As for prison gangs, while some are extension of criminal organisations active outside the prison, others exist and establish themselves in the isolation of the prison setting. For this reason, this study does not consider prison gangs, as they occur in a specific and institutionalised settings, and therefore individuals’ recruitment is influenced by different contextual factors (Blevins, Johnson Listwan, Cullen, & Lero Jonson, 2010; Wood, Alleyne, Mozova, & James, 2014). Furthermore, while there is a relevant literature on prison gangs, this field is mostly separate from the literature on OC, which emphasises the social embeddedness into the legitimate world. The exclusion of terrorist groups is due to the ideological/political motivation of such organisations. Furthermore, a Campbell systematic review on the factors leading to radicalisation and recruitment into terrorism is currently ongoing (Litmanovitz et al., 2017).

#### 3.1.3 Types of risk factors

This systematic review aims at identifying the risk factors associated to recruitment to OCGs. With regards to the measurement of the risk factors, we will only include measures taken at the individual level. Among the types of factors identified by our review, we expect to include demographic, social, economic, psychological and criminal history factors.

To consider a variable as a risk factor, the variable must occur prior to the outcome (Murray, Farrington, & Eisner, 2009). The risk factor therefore must precede the outcome, that is, OCG membership. Some factors, however, may be considered as preceding the outcome even if included in cross‐sectional studies, as they do not vary over the life course (e.g., sex and race). Some scholars argue that such time‐invariant factors cannot be considered as risk factors due to their fixed nature (see Murray et al., 2009). However, this systematic review will consider as risk factors for OCG membership not only those predictors resulting from longitudinal studies—measuring the factors preceding the occurrence of the outcome—but also time‐invariant factors estimated from cross‐sectional studies. Self‐reported retrospective data assessing risk factors preceding the outcome will also be considered, though they present some biases as they are based on individual's recall of past events (Murray et al., 2009). This choice is driven by the goal to include as many studies as possible given the lack of any systematic review on the recruitment into OC. Due the difficulties of collecting longitudinal data on OCGs, we expect to find few longitudinal studies on OCG membership (see Bruinsma, 2015).

We recognise the difficulty of establishing causation for risk factors deriving from observational designs. We acknowledge that the option may cause some factors to be measured only after the recruitment into OCGs has already occurred (e.g., unemployment, low education). In line with previous systematic reviews (Higginson et al., 2018; Klein & Maxson, 2006), this systematic review will attempt to classify as *predictors* the risk factors measuring conditions preceding the recruitment into OCGs and as *correlates* the risk factors measuring conditions occurring simultaneously or after the recruitment. Effects for predictors and correlates will be reported separately.

#### 3.1.4 Types of outcome measures

The outcome of interest in this systematic review is the recruitment into OCGs. As discussed in Section 1, recruitment refers to the different processes leading individuals to the stable involvement into OCGs. We will not differentiate among different forms of recruitment to OCGs. Therefore, we will include studies considering recruitment, affiliation and other forms of stable involvement. If relevant, the impact the risk factors on different forms of recruitment will be analysed through a moderator analysis.

The systematic review includes only studies that measure recruitment into OCGs at the individual level, measured with either a dichotomous or a categorical variable. In the case of a categorical variable (e.g., OCG membership, former membership, nonmembership, etc.), separate meta‐analyses will be carried out for each paired OCG–non‐OCG for which effect sizes can be extracted (e.g., OCG membership vs. former membership), with the outcomes being compared and discussed in the review.

The review will include self‐reported, peer‐reported, practitioner‐reported and police‐reported measures of individual OCG membership. If applicable, we will assess heterogeneity due to measurement methods with moderator analysis.

### 3.2 Search methods

#### 3.2.1 Search terms

**Figure 1:**
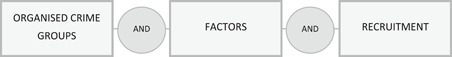
Query structure

This review relies on a threefold query structure that ensures systematic, thorough and efficient results. The queries incorporate all aspects that are relevant to the risk factors relating to the recruitment into different types of OCGs. The search terms from each of the three main categories (i.e., OCGs, factors and recruitment) combined formed the queries (Figure 1). The Boolean Operator “OR” connected keywords pertaining to the same category, while the Boolean Operator “AND” connected keywords from different categories (see Table A1 in Appendix A). This query structure ensured to retrieve all the studies containing at least one term from each word category.

#### 3.2.2 Search locations and languages

Given the transdisciplinary approach of this systematic review, the search for relevant studies relies on 12 databases relating to different research disciplines.^2^ The suitable studies encompass academic and grey literature written in English, French, German, Italian and Spanish, and pertaining to social, psychological and economic disciplines.^3^ No limitations apply as to their year of publication or geographic origin. Both academically published and grey literature is being considered. Table 1 reports the list of databases indicating in which language the search was conducted and which search technique was applied. When available, the preferred technique was to search title, abstract and keywords.

**Table 1:** List of databases and search techniques

Language	Database	Sub‐database	Search technique
English	EBSCO	Criminal Justice Abstracts	Abstract
	Open Grey		Full‐text
	ProQuest	Social Sciences Premium	Abstract
		NJCRS	
		PsycInfo	
		Abi/Inform	
		International Bibliography of the Social Sciences	
		Public Health Database	
		Military Database	
		EconLit	
		PsycArticles	
	PubMed		Title and abstract
	Scopus		Title, abstract and keyword
	Web of Science		Title
French	Google Scholar		Full‐text
	Sudoc.Abes		Title
German	Sowiport		Title
Italian	Riviste Web		Full‐text
Spanish	Liliacs		Title, abstract and subject
	ProQuest	Latin America and Iberia database	Full‐text

To validate the search terms and queries, the research team attended two meetings with a librarian to ensure the inclusion of all databases relevant to this systematic review. Table A3 in Appendix A shows the list of databases and the related queries used to perform the research (Table A4).

#### 3.2.3 Multistage approach to searching

Apart from identifying relevant literature through scientific databases, researchers will also contact experts to receive suggestions on relevant studies that may not have been included in the systematic review yet. First, several renown authors in the field of OCGs will be contacted: Jay Albanese (Virginia Commonwealth University, USA), Paolo Campana (University of Cambridge, UK), Scott Decker (Arizona State University, USA), Edward Kleemans (Vrije University of Amsterdam, NL), Klaus Von Lampe (John Jay College of Criminal Justice, USA), Carlo Morselli (University of Montreal, CA), Arthur Lurigio (Loyola University Chicago), Letizia Paoli (Katholieke Universiteit Leuven, BE), David Pyrooz (University of Colorado Boulder, USA), Sonja Wolf (Centro de Investigación y Docencia Económicas, MEX). Second, this list of experts will be implemented on the basis of the screening of the literature done by this systematic review. More precisely, the authors of the literature included after the full text screening will be also contacted.

The research team will further identify relevant literature from the bibliographies of the studies that will be selected for full‐text screening. As for the selection of studies from the database searches, these additional studies will be assessed for full‐text eligibility.

### 3.3 Selection of studies

#### 3.3.1 Preparatory activities

The review process will incorporate all the studies retrieved through database search. Metadata for each study will be imported to the Covidence online platform, which provides an environment to manage and conduct systematic reviews.^4^

After the removal of duplicate entries, the research team will be trained for the screening of relevant studies. The training will include a comprehensive briefing on the purpose and scope of the systematic review, followed by a tentative screening phase during which each reviewer will independently conduct the title‐and‐abstract screening of a set of 100 studies. The results will then be discussed among all researchers to reveal divergent interpretations and other issues, and maintain common criteria for the inclusion of studies in the systematic review.

To ensure reliability, throughout the screening process two reviewers will screen each document. A third researcher will settle divergent screening decisions, where necessary in consultation with the full review team.

#### 3.3.2 Eligibility screening criteria

As a first step, the screening will be based on the information reported in titles and abstracts. If the document is relevant in light of the aim of this systematic review, that is, investigates recruitment into OCGs as main aim of the study, it will be filtered in. If the document is irrelevant, it will be filtered out. If the information report in the title and abstract do not allow to include/exclude the document, the study will be kept for full‐text screening. In other words, we will keep every study that cannot be dropped, rather than the other way around.

As a second step, the screening will be based on the information reported in the full‐text.^5^ Each document will have to meet all the eligibility criteria listed in the “Eligibility screening form” (see Appendix B). The “Eligibility screening form” will guide the selection process by including only empirical documents that are focused on OGCs as defined in the paragraph “Types of organised crime groups”, examining clearly defined factors leading to recruitment into OCGs at an individual level. If the document meets all the eligibility criteria, it will be filtered in. If none of the eligibility criteria can be definitively answered in the positive based on the full‐text screening, the study will be filtered out. While in the previous phase we have favoured inclusivity, in this phase every criterion needs to be conclusively met, on penalty of study exclusion.

#### 3.3.3 Study coding

The quantitative, mixed‐method and qualitative studies that met all full‐text screening criteria will be independently coded by two reviewers based on a detailed coding guide (see Appendix B). Mixed‐method studies will be coded two times, one each for their empirical qualitative and quantitative sections. Item‐based questionnaire‐style coding documents have been used in previous reviews (e.g., Higginson et al., 2018). Types of OCGs will be initially coded into different categories, that is, mafias, drug trafficking groups, adult gangs and outlaw motorcycle gangs, and a residual category of other criminal groups (see items 12 and 13 of Table A4 in Appendix C). Such categories may be redefined based on the types of OCGs addressed by included studies and will also serve to conduct moderator analysis. The results will be compared and any coding conflict will be resolved through exchanges with a third reviewer.

### 3.4 Quality assessment

A large section of our coding protocol intends to assess the risk of study bias for quantitative or mixed‐method studies (questions 58–85 in Appendix C). This section will allow us to investigate a large variety of potential issues the studies in our review may have with sample selection, risk factors and outcome definition and application and statistical modelling, including diagnostic measures on the statistical models. Importantly, it will allow us to analytically reach an overall risk‐of‐bias rating for each study in our review. The quality assessment section is largely an adaptation of Higginson and colleague's systematic review (Higginson et al., 2018) and of PROBAST risk‐of‐bias tool for prediction models (PROBAST, 2018). We will interpret overall risk of bias as follows:

**Table:** 

Overall risk of bias judgement	
Low risk of bias	If all domains were rated low risk of bias.
High risk of bias	If at least one domain is judged to be at high risk of bias.
Unclear risk of bias	If an unclear risk of bias was noted in at least one domain and it was low risk for all other domains.

(Adapted from PROBAST, 2018, p. 8)

In keeping with previous meta‐analysis protocols, we will not exclude low‐quality studies (see Higginson et al., 2018). However, we will conduct moderator analysis to assess the effect of low‐quality studies on effect sizes. The results will be presented with the “traffic light” model adopted by De Vibe, Bjoerndal, Tipton, Hammerstroem, and Kowalski (2012).

Quality assessment on qualitative studies will be performed with the CASP Qualitative Checklist (Critical Appraisal Skills Programme, 2018). Regarding qualitative studies and their usage in our review, please see the “Treatment of qualitative research” section.

### 3.5 Statistical procedures

#### 3.5.1 Effect size metric and calculations

To perform the formal meta‐analysis, the different statistical measures reported in the quantitative and mixed‐method studies must be transformed into comparable effect size measures. If effect sizes are not directly included in the studies, we will extrapolate them based on reported statistics. Our coding document contains a subsection to help with this process (see items 35–57 in Appendix C). If the studies do not contain the necessary data for effect size extrapolation we will contact the authors of the studies.

We will code all effect sizes from our screened studies. Thanks to the coding guide, we will be able to group them based on several dimensions relevant for synthesis and interpretation. In particular, each effect size will be coded based on its document of origin, the nature of the two groups the effect was assessed on (e.g., OCG affiliates for the OCG and general criminals for the non‐OCG), and the risk factor it refers (items 1–4, 18–19 and 35 of our coding guide, respectively). We will carry out the statistical synthesis for all the comparable effect sizes between similar pairs of groups. Risk factors will also be classified based on their focus domain (sociodemographic, psychological, etc.) for easier comparation, synthesis and presentation (see item 36 in our coding guide).

Effect sizes can be calculated using three categories of statistics: Group means, for continuous variables; risk‐based association measures between two binary variables; and correlation measures between two either continuous, ordinal or categorial variables. We expect studies in our review to report their results using mainly group means differences and standard deviations for continuous variables, odds ratios for binary variables, and correlation measures, such as Pearson's correlation or regression coefficients. These three different forms of data will be transformed into effect sizes in the form of *log odds ratios* in order to perform meta‐analysis.

The logic of using log odds ratios as a common statistic is twofold. First, both odds ratios and log odds ratios are symmetrical across the two variables they reference. We expect the studies in our review to often consider OCG recruitment as an independent variable and what we would call a risk factor as dependent variable, in particular when reporting the difference in a continuous variable between an OCG group and a non‐OCG. Once the same statistical information is transformed into an odds ratio, the issue of directionality disappears: We can interpret the resulting effect size as the likelihood of OCG recruitment between groups with and without the risk factor, as intended for review.

Second, log odds ratios have the property of symmetry around their null value. While odds ratios are defined between 0 and positive infinity with a null value of 1 and asymmetrical standard errors, log odds ratios “normalize” the null value to 0 and are defined between negative infinity and positive infinity, with symmetrical standard errors regardless of sign (see Borenstein, Hedges, Higgins, & Rothstein, 2009, p. 35). This makes it easier to use them for analysis.

The conversion to log odds ratios entails, respectively:

1. For continuous variables for which group means and variance are reported, calculating first

- Cohen's *d*:

$$d=\frac{{\overset{®}{x}}_{\text{OC}}-{\overset{®}{x}}_{\text{NOC}}}{\sqrt{\left(\frac{\left(n_{\text{OC}}-1\right)s_{\text{OC}}^{2}+\left(n_{\text{NOC}}-1\right)s_{\text{NOC}}^{2}}{n_{\text{OC}}+n_{\text{NOC}}-2}\right)}}$$

- ***d*** 's standard error ***SE***_***d***_:

$${\mathrm{SE}}_{d}=\sqrt{\left(\frac{n_{\text{OC}}+n_{\text{NOC}}}{n_{\text{OC}}n_{\text{NOC}}}+\frac{d^{2}}{2\left(n_{\text{OC}}+n_{\text{NOC}}\right)}\right)}$$

Where

**Table:** 

${\overset{®}{x}}_{\text{OC}}$	Mean value of the variable of interest in the OC sample
${\overset{®}{x}}_{\text{NOC}}$	Mean value of the variable of interest in the non‐OC sample
$n_{\text{OC}}$	OC sample size
$n_{\text{NOC}}$	non‐OC sample size
$s_{\text{OC}}^{2}$	Variance of the variable of interest in the OC sample
$s_{\text{NOC}}^{2}$	Variance of the variable of interest in the non‐OC sample

These measures will then be used to calculate:

- Log odds ratio:

$$\mathrm{log OR}=\frac{\pi d}{\sqrt{3}}$$

- Log OR standard error:

$${\mathrm{SE}}_{\mathrm{log OR}}=\frac{\pi {\text{SE}}_{d}}{\sqrt{3}}$$

2. For binary variables for which contingency tables or odds ratios are reported, calculating:

- Log odds ratio:
$$\mathrm{log OR}=\mathrm{ln OR}=\ln\left(\frac{n_{1}^{\mathrm{OC}}n_{0}^{\mathrm{NOC}}}{n_{0}^{\mathrm{OC}}n_{1}^{\mathrm{NOC}}}\right)$$
- Log OR standard error:

$${\mathrm{SE}}_{\mathrm{log OR}}=\sqrt{\frac{1}{n_{1}^{\mathrm{OC}}}+\frac{1}{n_{0}^{\mathrm{OC}}}+\frac{1}{n_{1}^{\mathrm{NOC}}}+\frac{1}{n_{0}^{\mathrm{NOC}}}}$$

Where

**Table:** 

$n_{1}^{\text{OC}}$	Number of OC individuals with the variable of interest
$n_{0}^{\mathrm{OC}}$	Number of OC individuals without the variable of interest
$n_{1}^{\mathrm{NOC}}$	Number of non‐OC individuals with the variable of interest
$n_{0}^{\mathrm{NOC}}$	Number of non‐OC individuals without the variable of interest

3. For continuous variables for which only Pearson's correlation is reported, calculating:

- ***r's standard error SE***_***r***_:
$${\mathrm{SE}}_{r}=\frac{1-r^{2}}{\sqrt{n-1}}$$
  Then, using **r** and ***SE***_***r***_, calculating
- ***Cohen's d***:

$$d=\frac{2r}{\sqrt{1-r^{2}}}$$

- ***d*'s standard error SE** *_**d**_*:

$${\mathrm{SE}}_{d}=\frac{2{\mathrm{SE}}_{r}}{{\left(1-r^{2}\right)}^{2}}$$

And finally, using *d* and its *SE*_*d*_ to calculate the *log OR* and its *SE*_*log OR*_ using the same formulas used for continuous variables for which group means were reported.

Another source of effect sizes for review are coefficients from regression models reported in our studies. As regression coefficients are sensitive to the set of covariates the models use, they need to be adjusted before analysis in order to remove covariate effect. For OLS regression models, this entails calculating the semi‐partial correlation ***r***_***sp***_, which we will do following procedures suggested by Aloe and Thompson (2013). We will then calculate the log odds ratios following the same procedure used for “regular” product–moment correlations. In logistical regression models, regression coefficients are already presented as log odds ratios. In this case we will simply code them as they are, together with their standard error, to be directly used in meta‐analysis.

**Figure 2:**
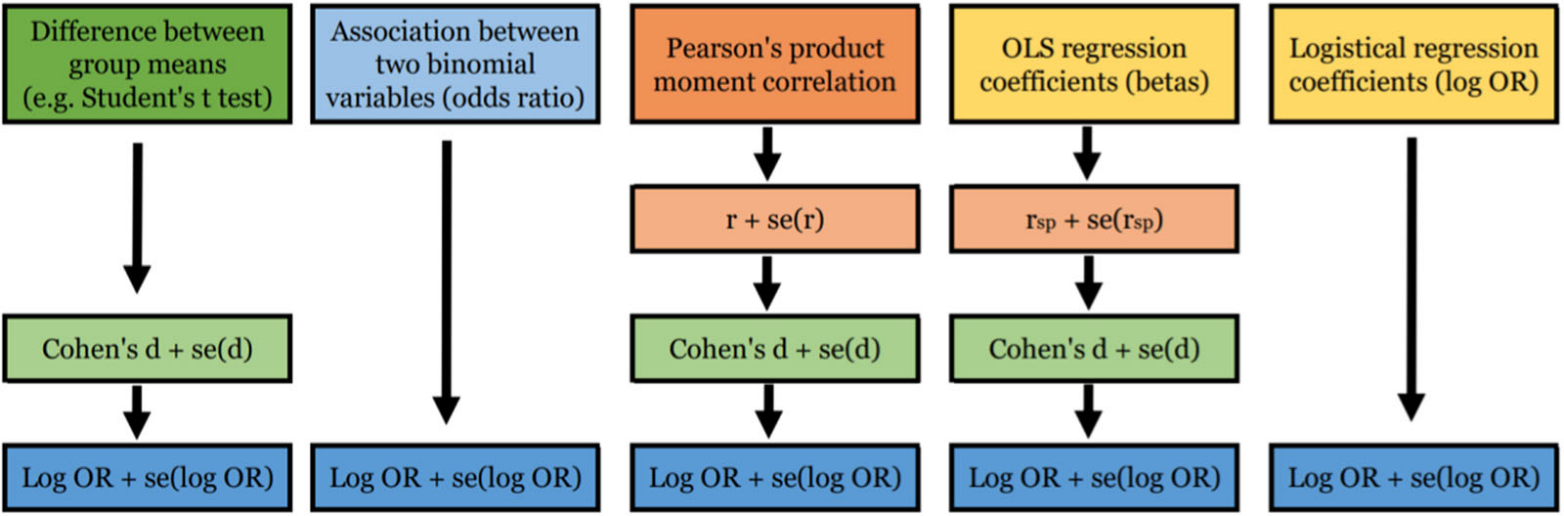
Effect sizes extraction by type of relation. [Color figure can be viewed at wileyonlinelibrary.com]

Figure 2 synthetically represents how effect size extraction and conversion will be carried out.

#### 3.5.2 Method of synthesis

If at least two studies provide effect sizes for the same predictor or correlate, we will conduct a random‐effects meta‐analysis on that factor using inverse variance weighting. This way we will calculate the overall weighted mean effect estimate of each separate factor on OCG recruitment. The result will be presented in a forest plot with 95% confidence intervals. In keeping with previous reviews (Hawkins et al., 2000; Higginson et al., 2018), we will carry out meta‐analysis using log odds ratios, then convert the results into odds ratios for presentation. As each effect size will refer to the pairing of an OCG and a non‐OCG (e.g., involved in an OCG vs. general criminals), we will only carry out meta‐analyses among effect sizes that measure the same factor for the same group pairing. On the other hand, meta‐analyses that refer to the same factor across different OCG and non‐OCG pairings will be presented in the same forest plot but not further synthesized.

If the paucity of the studies analysing a factor prevents us from completing a formal meta‐analysis for that factor, we will present forest plots and confidence intervals for each factor without attempting a statistical synthesis. This may be the case of studies reporting on factors similar in nature but for which a meta‐analytic synthesis would be hardly meaningful (e.g., unemployment and low socioeconomic conditions).

#### 3.5.3 Assessment and investigation of heterogeneity

While the main scope of a meta‐analysis is to assess the global effect of a factor on a given phenomenon with a degree of precision superior to that of any single study, the study of heterogeneity can provide indications on how to interpret that effect (while quantitatively describing the efficacy of the calculated global effect size; Borenstein et al., 2009). For instance, a small degree of heterogeneity emerging when comparing otherwise similar studies made on different populations tells us that population differences do not appear to play a large part on that factor‐outcome association—a finding which may otherwise have flown under the radar. For this reason, it is useful both to attempt meta‐analyses between studies we suspect to have some degree of methodological incomparability, and to give a statistical value to that incomparability. To this end, we will assess heterogeneity between studies with the *Q*, *I* ^2^, *τ* and *χ* ^2^ statistics.

#### 3.5.4 Sensitivity and subgroup analysis

If enough studies are available, we are going to conduct sensitivity analyses to further assess the effect of study heterogeneity and risk level on the results of the review. In particular, the relevant subgroups of studies are going to be selected based on bias risk (as assessed in questions 58–85 of our coding document, see Appendix C) and geographic scope of the study. We may also choose to conduct complete subgroup analyses on the same or different subgroups to formally explore how the study variables the group division was based on impact global effect sizes.

#### 3.5.5 Assessment of publication bias

To assess potential publication bias in each subgroup we will use funnel plots, a specialised form of scatter plots used in meta‐analysis to visually identify publication and other bias (Sterne, Becker, & Egger, 2006) Furthermore, publication bias will be adjusted with trim and fill analysis, aiming to “both identify and correct for funnel plot asymmetry arising from publication bias” (Higgins & Green, 2011). These steps will follow the methodology suggested by Rothstein, Sutton, and Borenstein (2005).

### 3.6 Treatment of qualitative research

This systematic review will include not only quantitative studies but also qualitative ones, as qualitative research is particularly relevant in the field of study of OCGs. Systematic reviews have generally excluded qualitative studies because of the impossibility of using their findings to draw conclusions. Nonetheless, Campbell policies and guidelines have recently opened up to the inclusion of qualitative and descriptive research, which can provide a more comprehensive overview of the object of study.

Qualitative studies will be systematically retrieved and screened for inclusion. They will be coded together with the quantitative literature. The final part of coding for qualitative studies includes their quality assessment, which will be carried out based on the CASP Qualitative Checklist (Critical Appraisal Skills Programme, 2018). The studies obtaining a positive evaluation on the basis of questions 1–9 of the CASP Qualitative Checklist will be used to inform and contextualize the evidence and findings of the quantitative studies.

## ROLES AND RESPONSIBILITIES

- Content: Francesco Calderoni, Elisa Superchi, Tommaso Comunale, Gian Maria Campedelli, Martina Marchesi, Niccolò Frualdo
- Systematic review methods: Francesco Calderoni, Tommaso Comunale, Gian Maria Campedelli, Martina Marchesi
- Statistical analysis: Francesco Calderoni, Gian Maria Campedelli, Niccolò Frualdo
- Information retrieval: Tommaso Comunale, Gian Maria Campedelli, Martina Marchesi

## SOURCES OF SUPPORT

This review is being conducted as part of PROTON (Modelling the PRocesses leading to OC and TerrOrist Networks), a European Commission funded project within the Horizon 2020 programme (Grant Agreement: 699824).

## CONFLICT OF INTERESTS

None of the authors has previously been involved in relevant interventions or has published other reviews on the topic. This systematic review is being conducted as part of PROTON project, as stated in the section above. Other two systematic reviews are conducted within the project but they will be related to other topics.

## PRELIMINARY TIMEFRAME

**Table:** 

Search for eligible studies	September–October 2019
Training and pilot testing on screening criteria	November 2019
Screening the results from the literature search	November–December 2019
Relevance assessments and coding of eligible studies	January 2020
Extraction of data from included studies	January 2020
Preliminary exploration of statistical analysis	February 2020
Preparation of the final review report	March 2020

Plans for updating the review

The authors plan to update the review every 5 years.

## AUTHOR DECLARATION

### Authors’ responsibilities

By completing this form, you accept responsibility for preparing, maintaining and updating the review in accordance with Campbell Collaboration policy. The Campbell Collaboration will provide as much support as possible to assist with the preparation of the review.

A draft review must be submitted to the relevant Coordinating Group within 2 years of protocol publication. If drafts are not submitted before the agreed deadlines, or if we are unable to contact you for an extended period, the relevant Coordinating Group has the right to deregister the title or transfer the title to alternative authors. The Coordinating Group also has the right to deregister or transfer the title if it does not meet the standards of the Coordinating Group and/or the Campbell Collaboration.

You accept responsibility for maintaining the review in light of new evidence, comments and criticisms, and other developments, and updating the review at least once every 5 years, or, if requested, transferring responsibility for maintaining the review to others as agreed with the Coordinating Group.

### Publication in the Campbell Library

The support of the Coordinating Group in preparing your review is conditional upon your agreement to publish the protocol, finished review, and subsequent updates in the Campbell Library. The Campbell Collaboration places no restrictions on publication of the findings of a Campbell systematic review in a more abbreviated form as a journal article either before or after the publication of the monograph version in Campbell Systematic Reviews. Some journals, however, have restrictions that preclude publication of findings that have been, or will be, reported elsewhere and authors considering publication in such a journal should be aware of possible conflict with publication of the monograph version in Campbell Systematic Reviews. Publication in a journal after publication or in press status in Campbell Systematic Reviews should acknowledge the Campbell version and include a citation to it. Note that systematic reviews published in Campbell Systematic Reviews and co‐registered with the Cochrane Collaboration may have additional requirements or restrictions for co‐publication. Review authors accept responsibility for meeting any co‐publication requirements.

## Footnotes

1 Developmental and life‐course criminology, term coined by Farrington (2003), is concerned with key factors for offending, effects of life events and life transitions on offending and development of offending.

2 The research team obtained temporary access to two specific sub‐databases, that is, National Criminal Justice Reference Service and Latin America and Iberia Database.

3 It was decided to exclude studies in Dutch since during multiple contacts with Dutch scholars confirmed that most of the studies published by Dutch scholars in this field are also indexed and published in English.

4 The Covidence platform is a core component of Cochrane's review production toolkit improving the production of systematic reviews. It allows to import citations of the studies included in the systematic review and fasten the screening phase by enabling the members of the review team to collaborate and perform the double‐checked screening simultaneously keeping track of all passages.

5 The studies deemed suitable for full‐text screening will be retrieved and evaluated. The research team will directly request a copy of the documents that are not available for download to the authors or publishing institutions. A second training will be held to ensure consistency in performing full‐text screening between reviewers. Each reviewer will be assigned the same sample of documents (*n* = 20) and results will be compared within the research team before moving on to the actual screening.

## APPENDIX A

Table A1 and A2

**Table A1:** Search categories and related search terms

Search category	Search terms
Organised Crime Group	Criminal organisation
	Criminal organization
	Criminal association
	Organized crime
	Organised crime
	Mafia
	Crim* network*
	dto*
	Drug trafficking organ*
	Motorcycle gang*
	Bikie gang*
	Crim* group*
	Crim* cartel
Factor	Risk factor*
	Predictor*
	Driver*
	Determinant*
	Correlate*
Recruitment	Involv*
	Recruit*
	Starter*
	Affiliat*
	Membership
	Criminal career*
	Criminal trajector*

**Table A2:** Databases and related queries

Database	Query
EBSCO	AB (("criminal organisation" OR "criminal organization" OR "criminal association" OR "organized crime" OR "organised crime" OR mafia OR "crim* network*" OR dto* OR "drug trafficking organ*" OR "drug cartel*" OR "motorcycle gang*" OR "bikie gang*" OR "crim* group*" OR "crim* cartel")) AND AB ((involv* OR starter* OR affiliat* OR membership OR recruit* OR "criminal career*" OR "criminal trajector*")) AND AB (("risk factor*" OR predictor* OR driver* OR determinant* OR correlate*))
Open Grey	("criminal organisation" OR "criminal organization" OR "criminal association" OR "organized crime" OR "organised crime" OR mafia OR "crim* network*" OR dto* OR "drug trafficking organ*" OR "drug cartel*" OR "motorcycle gang*" OR "bikie gang*" OR "crim* group*" OR "crim* cartel") AND (involv* OR starter* OR affiliat* OR membership OR recruit* OR "criminal career*" OR "criminal trajector*") AND ("risk factor*" OR predictor* OR driver* OR determinant* OR correlate*) NOT(narcosis OR ganglion* OR narcolept* OR marathon* OR organ* OR maraviroc* OR gangetic* OR gangue OR "marangoni" OR narcoleps* OR ganger OR mafic OR maranh*) lang:"en"
ProQuest (English)	AB("criminal organisation" OR "criminal organization" OR "criminal association" OR "organized crime" OR "organised crime" OR mafia OR "crim* network*" OR dto* OR "drug trafficking organ*" OR "drug cartel*" OR "motorcycle gang*" OR "bikie gang*" OR "crim* group*" OR "crim* cartel") AND AB(involv* OR starter* OR affiliat* OR membership OR recruit* OR "criminal career*" OR "criminal trajector*") AND AB("risk factor*" OR predictor* OR driver* OR determinant* OR correlate*)
PubMed	("organized crime"[Title/Abstract] OR "organised crime"[Title/Abstract] OR "criminal organization"[Title/Abstract] OR "criminal organisation"[Title/Abstract] OR "mafia"[Title/Abstract] OR "drug trafficking organization"[Title/Abstract] OR "drug trafficking organisation"[Title/Abstract]) AND (recruitment[Title/Abstract] OR affiliation[Title/Abstract] OR membership[Title/Abstract] OR "risk factor" [Title/Abstract] OR predictor[Title/Abstract] OR correlate[Title/Abstract])
Scopus	(TITLE‐ABS‐KEY (("organised crime" OR "organized crime" OR "criminal organization" OR "criminal organisation" OR "mafia" OR "drug trafficking organization" OR "drug trafficking organisation")) AND TITLE‐ABS‐KEY ((recruitment OR affiliation OR membership "risk factor" OR predictor OR correlate)) AND NOT TITLE‐ABS‐KEY ((gangl OR narcosis OR narcolept OR marathon OR organ OR organs OR maraviroc OR gangetic))) AND (LIMIT‐TO (SUBJAREA, "SOCI") OR LIMIT‐TO (SUBJAREA, "MEDI") OR LIMIT‐TO (SUBJAREA, "PSYC") OR LIMIT‐TO (SUBJAREA, "ARTS") OR LIMIT‐TO (SUBJAREA, "ECON") OR LIMIT‐TO (SUBJAREA, "BUSI") OR LIMIT‐TO (SUBJAREA, "NURS") OR LIMIT‐TO (SUBJAREA, "NEUR") OR LIMIT‐TO (SUBJAREA, "HEAL")) AND (LIMIT‐TO (SRCTYPE, "j") OR LIMIT‐TO (SRCTYPE, "b") OR LIMIT‐TO (SRCTYPE, "k") OR LIMIT‐TO (SRCTYPE, "p") OR LIMIT‐TO (SRCTYPE, "d"))
Web of Science	(TI=("criminal organisation" OR "criminal organization" OR "criminal association" OR "organized crime" OR "organised crime" OR mafia OR "crim* network*" OR dto* OR "drug trafficking organ*" OR "drug cartel*" OR "motorcycle gang*" OR "bikie gang*" OR "crim* group*" OR "crim* cartel") AND TI=(involv* OR starter* OR affiliat* OR membership OR recruit* OR "criminal career*" OR "criminal trajector*") AND TI=("risk factor*" OR predictor* OR driver* OR determinant* OR correlate*)) AND LANGUAGE: (English); Indexes=SCI‐EXPANDED, SSCI, A&HCI, CPCI‐S, CPCI‐SSH, BKCI‐S, BKCI‐SSH, ESCI, CCR‐EXPANDED, IC Timespan=All years
Google Scholar	(milieu OR organisat* criminelle* OR criminalité organisée OR criminels organisés OR cartel criminel OR mafia) AND (facteur* OR risq* OR recrut*)
Sudoc.Abes	(milieu OR organisat* criminelle* OR criminalité organisée OR criminels organisés OR cartel criminel OR mafia) AND (facteur* OR risq* OR recrut*)
Sowiport	(“organisierte kriminalität” OR kriminelle* organisation* OR kriminelle* vereinigung* OR kriminelle* kartell* OR mafia* OR mafiaähnlich* OR motorradclub*) AND (OR faktor* OR prädiktor*)
Liliacs	(mafia OR "grupo criminal" OR "asociacion criminal" OR "crimen organizado" OR cartel OR "delincuencia organizada") AND (riesgo OR reclutamiento OR "carrera criminal" OR factor)
ProQuest (Spanish)	(mafia OR "grupo criminal" OR "asociacion criminal" OR "crimen organizado" OR cartel OR “delincuencia organizada") AND (riesgo OR reclutamiento OR "carrera criminal" OR factor)
Riviste Web	("crimine organizzato" OR "criminalità organizzata" OR "associazione delinquere" OR mafia OR "organizzazione criminale") AND (reclut* OR affilia* OR fattor* OR rischi* OR carriera)

## APPENDIX B ELIGIBILITY SCREENING FORM

Table A3

**Table A3:** Eligibility screening form

1. Does the document report on the OCGs as defined in this review?	0 = No
	1 = Yes
	99 = Can't tell
	*If no then stop*
2. Does the document investigate recruitment into OCGs as one of its main objectives?	0 = No
	1 = Yes
	99 = Can't tell
	*If no then stop*
3. Does the document make any empirical contribution to the study of the recruitment into OCGs?	0 = No
	1 = Yes
	99 = Can't tell
	*If no then stop*
4. Does the study discuss sufficiently well‐defined factor leading to recruitment into OCGs? For quantitative studies in particular, does each factor measure a single, reasonably defined characteristic?	0 = No
	1 = Yes
	99 = Can't tell
	*If no then stop*
5. Are factors of recruitment into OCGs assessed on an individual level?	0 = No
	1 = Yes
	99 = Can't tell
	*If no then stop*
6. If the document follows a quantitative or mixed‐method approach, does the study design allow to capture a sufficient variability between OCG members and non‐OCG members?	0 = No
	1 = Yes
	99 = Can't tell
	*If no then stop*

## APPENDIX C Document Coding Protocol

Table A4

**Table A4:** Document coding protocol (all documents)

Section		Variable	Value
Reference information	1	Study ID	
	2	Study authors	
	3	Study title	
	4	Publication year	
	5	Reference type	a. Peer reviewed journal article b. Book c. Book chapter d. Thesis or dissertation e. Other: _____
	6	Complete APA reference	
Study details	7	Language	a. English b. Spanish c. Italian d. French e. German
	8	Geographic scope	World region/Country
	9	Data source	a. Compiled by researcher (eg survey) b. Publicly available database: _____ c. Judicial records: _____ d. Investigative/police files: _____ e. Other: _____
	10	Research period	a. Start: _____ b. Finish: _____
	11	Ethical issues	a. N b. Y: _____
	12	Type of OCG	a. Mafia b. DTO c. Adult gang d. Outlaw motorcycle gang e. Other OCG: _____
	13	OCG name	____________________
	14	Study methodology	a. Quantitative b. Mixed method c. Qualitative

If the study is classified as “quantitative” go to Table A5.

**Table A5:** Coding protocol (only quantitative and mixed methods studies)

**Study methodology**	15	Type of observational study, if applicable	a. Longitudinal b. Cross‐sectional c. Case control d. NA
	16	Is the data source the same for the OCG and non‐OCG groups?	a. Y b. N c. Unclear
	17	If not, what is the data source for non‐OCG group(s)?	a. Compiled by researcher (e.g. survey) b. Publicly available database: _____ c. Judicial records:____ d. Investigative/police files: _____ e. Other: _____
	18	Non‐OCG group(s) composition (check any applicable)	a. Previous OCG members b. Involved with (not formal affiliates of) an OCG c. Serious non‐OCG criminals d. General non‐OCG criminals e. Non‐criminal sample(s) (e.g., community sample) f. Other
	19	Measure of OCG recruitment	a. OCG membership b. OCG affiliation c. Involvement in OC d. Other
	20	Nature of OCG recruitment measure	a. Dichotomous b. Categorical
	21	Source of OCG recruitment measure	a. Self‐reported b. Official data (e.g., judicial/police) c. Other: _____
	22	Is OCG recruitment described in replicable detail?	a. Y b. N c. Unclear
	23	Total sample size	____________________
	24	Size of OCG group	____________________
	25	Size of non‐OCG group	____________________
	26	Sample gender	a. M: _____ b. F: _____ c. Mixed
	27	Sample SES	a. Low b. Average c. High d. Mixed
	28	Is the study population described in replicable detail?	a. Y b. N c. Unclear
	29	Statistical model(s) used (e.g. logistical modelling)	____________________
	30	Was (Were) the statistical model(s) internally or externally validated?	____________________
	31	Model validation method(s)	____________________
	32	Performance measures of the model(s)	____________________
	33	Was data missing on risk factors or outcomes?	a. Y b. N c. Unclear
	34	If yes, how was missing data dealt with?	____________________
**Risk factors**	35	Risk factor	____________________
	36	Risk factor category	a. Sociodemographic b. Economic status c. Criminal history d. Psychological e. Other
	37	Conceptual definition of risk factor	____________________
	38	Operational definition	____________________
	39	Source of risk factor measure	a. Self‐reported b. Official data (e.g., judicial/police) c. Other: ____
	40	Risk factor measured retrospectively	a. Y b. N c. Unclear
	41	Is the risk factor time‐invariant?	a. Y b. N. In this case in non‐longitudinal studies the factor will be classified as correlate c. Unclear
	42	Was the effect size reported?	a. Y b. N
	*If yes*:		
	43	Reported risk factor effect size	____________________
	44	ES standard error	____________________
	45	ES confidence intervals	____________________
	*If not, we will use available data to calculate it*:		
	46	Mean value (OCG and non‐OCG groups)	____________________
	47	Standard deviation (OCG and non‐OCG groups)	____________________
	48	Alternatively, unadjusted correlation coefficient	____________________
	49	Alternatively, standardized correlation coefficient	____________________
	50	Alternatively, unadjusted regression coefficient	____________________
	51	Alternatively, standardized regression coefficient	____________________
	52	If dichotomous, fraction of OCG and non‐OCG groups with risk factor	____________________
	53	n size of OCG and non‐OCG groups for risk factor	____________________
	54	Risk factor difference between OCG and non‐OCG groups	____________________
	55	Extrapolated risk factor effect size	____________________
	56	Extrapolated ES standard error	____________________
	57	Extrapolated ES confidence intervals	____________________
**Risk of study bias**	*a. Risk of bias due to sampling and setting*		
	58	Are all sample inclusion/exclusion criteria listed?	a. Y b. N c. Unclear
	59	What are the inclusion/exclusion criteria?	____________________
	60	Sample selection precedes OCG involvement?	a. Y b. N c. Unclear
	61	Initial response rate, if applicable	____________________
	62	Attrition rate, if applicable	____________________
	63	Were all participants inclusion and exclusion choices appropriate?	a. Y b. N c. Unclear
	64	Overall risk of bias due to sample selection?	a. Low b. High c. Unclear
	65	Rationale of bias rating:	
	*b. Risk of bias due to the risk factors or their assessment*		
	66	Were all risk factors described in replicable detail?	a. Y b. N c. Unclear
	67	Were risk factors defined and assessed in a similar way for all participants?	a. Y b. N c. Unclear
	68	Were all risk factors based on validated measures?	a. Y b. N c. Unclear
	69	Is there a pre‐measure for all risk factors (including obtained retrospectively)?	a. Y b. N c. Unclear
	70	Were confounding factors measured before OCG involvement (including obtained retrospectively)?	a. Y b. N c. Unclear
	71	Overall risk of bias due to risk factors or their assessment?	a. Low b. High c. Unclear
	74	Rationale of bias rating:	____________________
	*d. Risk of bias due to statistical procedures*		
	75	Was there a reasonable number of individuals in the sample?	a. Y b. N c. Unclear
	76	Were continuous and categorical risk factors statistically handled appropriately?	a. Y b. N c. Unclear
	77	If applicable, were all enrolled participants included in the analysis?	a. Y b. N c. Unclear
	78	Were missing data handled appropriately?	a. Y b. N c. Unclear
	79	Were complexities in the data (e.g. sampling of controls) accounted for appropriately?	a. Y b. N c. c. Unclear
	80	Overall risk of bias due to statistical analysis?	a. Low b. High c. Unclear
	81	Rationale of bias rating:	____________________
	*e. Overall study risk of bias*		
	82	Overall judgement of risk of bias	a. Low b. High c. Unclear
	83	Summary of sources of potential risk	____________________
	84	Overall judgement of study applicability to the research question	a. Low b. High c. Unclear
	85	Summary of applicability concerns	

If the study is classified as “qualitative”, go to the CASP Qualitative Checklist (Critical Appraisal Skills Programme, 2018) and code it based on the first nine items (i.e., exclude the last one).

If the study is classified as “mixed method”, Table A5 will be used for its empirical quantitative section and the CASP Qualitative Checklist will be used for its empirical qualitative section.
